# Impact on Patient’s Appearance Perception of Autologous and Implant Based Breast Reconstruction Following Mastectomy Using BREAST-Q

**DOI:** 10.1007/s00266-022-02776-z

**Published:** 2022-02-28

**Authors:** Paolo Persichetti, Mauro Barone, Rosa Salzillo, Annalisa Cogliandro, Beniamino Brunetti, Silvia Ciarrocchi, Mario Alessandri Bonetti, Stefania Tenna, Michail Sorotos, Fabio Santanelli Di Pompeo

**Affiliations:** 1 Faculty of Medicine and Psychology, U.O.D. Chirurgia Plastica, Sapienza University of Rome, Sant’Andrea Hospital, Via di Grottarossa, 1035-1039, 00189 Rome, Italy; 2 Plastic and Reconstructive Surgery Unit, Campus Bio-Medico University of Rome, Rome, Italy; 3 Research group “To be and to appear: Objective indication to Plastic Surgery” of Campus Bio-Medico University in Rome, Rome, Italy; 4 Institute of Philosophy of Scientific and Technological Activity, Campus Bio-Medico University of Rome, Rome, Italy; 5 Department of Plastic, Reconstructive and Aesthetic Surgery, I.R.C.C.S. Istituto Galeazzi, University of Milan, Milan, Italy; 6 Department of Medicine, Surgery and Dentistry “Scuola Medica Salernitana”, PhD School of Translational Medicine of Development and Active Ageing, Università degli Studi di Salerno, Salerno, Italy; 7 Plastic and Reconstructive Surgery Unit, Azienda Ospedaliera Sant’Andrea, Rome, Italy

**Keywords:** Patient satisfaction, Quality of life, BREAST-Q, Breast reconstruction, Diep flap, Implant-based reconstruction

## Abstract

**Introduction:**

The purpose of this study is to determine if there is a better quality of life with one of the two techniques and if the results are in line with those already present in the literature. The hypothesis from which we started is to demonstrate that cancer patients who undergo a deep inferior epigastric perforator flap (DIEP) breast reconstruction surgery are more satisfied and have a higher level of quality of life compared to those subjected to an intervention of reconstruction with prosthesis.

**Materials and Methods:**

All patients undergoing reconstruction from January 2010 to July 2018 were eligible for inclusion. This is a retrospective cohort study carried out using the patients of two plastic surgery departments who have undergone monolateral or bilateral implant-based or DIEP flap breast reconstruction. We administered BREAST-Q questionnaire electronically almost 2 year after surgery. Patients were divided into two groups: implant-based and autologous breast reconstruction with DIEP flaps. Baseline demographics and patient characteristics were analyzed using a Students *t*-test (continuous variables) or Chi-square/Fisher’s exact test (categorical variables). Mean standard deviation BREAST-Q scores were reported for the overall cohort and by modality for the postoperative period. The linear regression model was applied to all BREAST-Q score with all predictor factors.

**Results:**

Of the 1125 patients involved, only 325 met the inclusion criteria and were enrolled in this study; specifically, 133 (41%) DIEP and 192 (59%) prosthetic reconstructions. We summarized the results of the principal scales of BREAST-Q module: satisfaction with breast, psychosocial well-being, satisfaction with outcome, and sexual well-being in which the autologous group was always more satisfied. We reported results of all linear regression models with higher values for the DIEP group independently from predictors.

**Conclusion:**

This is the first study performed on the Italian population that compares autologous surgical techniques with the implantation of breast implants. In this population, DIEP is considered the technique that leads to the highest satisfaction in all BREAST-Q scores.

**Level of Evidence IV:**

This journal requires that authors assign a level of evidence to each article. For a full description of these Evidence-Based Medicine ratings, please refer to the Table of Contents or the online Instructions to Authors www.springer.com/00266.

## Introduction

The breasts represent the fulcrum of female sexuality and are one of the central and most important points for all women [1, 2]. It has been well known for decades that mastectomy involves not only a physical demolition, but also results in psychological discomfort in a woman’s social, relational, and sexual life [3, 4]. Over the decades, reconstructive surgery techniques have been increasingly refined in order to allow patients to have a high quality of life. The reconstructive technique must be chosen based on the characteristics of the patient, the therapies already performed or to be performed, and the tissue to be reconstructed [4–7]. However, we can evaluate in the long term and with the same initial condition and therapy, what is the percentage of the body of women who have undergone mastectomy and who have been reconstructed with microsurgical flaps and breast implants [8, 9]. Patient-reported outcomes following breast reconstruction are one of the most important success parameters. In this systematic review and meta-analysis, we aimed to compare the two methods using the recognized BREAST-Q questionnaire [3, 9]. In the literature, there are already comparative and prospective studies concerning this topic, all of which conclude that microsurgical reconstructions lead to the best long-term results, with fewer secondary procedures and with a better quality of life [10–13]. Many studies have been performed with generic evaluation scales, with ad hoc questionnaires, and others with specific questionnaires. BREAST-Q is currently the most complete questionnaire and is indicated as the best tool for postoperative evaluation of breast interventions [14]. Few studies have used BREAST-Q. In Italy, there is no study that compares the two long-term reconstructive techniques using the BREAST-Q. For this reason, the purpose of this study is to determine if there is a better quality of life with one of the two techniques and if the results are in line with those already present in the literature. The hypothesis from which we started is to demonstrate that cancer patients who undergo a deep inferior epigastric perforator flap (DIEP) breast reconstruction surgery are more satisfied and have a higher level of quality of life compared to those subjected to an intervention of reconstruction with prosthesis.

## Materials and Methods

An institutional review board approved this study, which was performed to evaluate PROs (patients reported outcomes) in post-mastectomy breast reconstruction and which were assessed as a component of routine clinical care. All patients undergoing reconstruction from January 2010 to July 2018 were eligible for inclusion.

This is a retrospective cohort study carried out using the patients of two plastic surgery departments who have undergone monolateral or bilateral implant-based (Campus Bio-Medico University Hospital of Rome) or DIEP flap breast reconstruction (Sant’Andrea University Hospital of Rome). The BREAST-Q PROM (patients reported outcome measures) was administered postoperatively almost 2 years from the last surgical procedure. Patients were divided into two groups: implant-based and autologous breast reconstruction with DIEP flaps. Inclusion criteria consisted of patients who underwent to breast reconstruction for cancer, had a follow-up of at least 2 years, were fluent in the Italian language, and signed the study consent. Patients having undergone prophylactic mastectomy due to genetic indication from deleterious BRCA1/2 or CDH1 mutations were also included in the study. Patients were excluded if they underwent delayed procedures, had a follow-up of less than 2 years, had postoperative complications that compromised reconstruction, and were legally incompetent, as well as women who did not sign the consent form to participate to this study. Patient responses were recorded on-site, either electronically or physically. Demographic data, treatment method, and postoperative outcomes were recorded secondarily. Variables recorded for each patient included age, body mass index (BMI), history of smoking, preoperative/postoperative breast irradiation, neoadjuvant/adjuvant chemotherapy, diabetes, hypertension, and timing. Baseline demographics and patient characteristics were analyzed using a Students *t*-test (continuous variables) or Chi-square/Fisher’s exact test (categorical variables). Mean standard deviation (SD) BREAST-Q scores were reported for the overall cohort and by modality for the postoperative period. The linear regression model was applied to all BREAST-Q score with all predictor factors. Linear regression attempts to model the relationship between two variables by fitting a linear equation to observed data. One variable is considered to be an explanatory variable, and the other is considered to be a dependent variable.

### BREAST-Q

BREAST-Q [15], published in 2009, is a rigorously developed and validated breast surgery-specific PRO-instrument. It has been used to evaluate over 22,000 women who had different types of breast surgery. Development of the BREAST-Q conceptual framework and scale set involved the literature review, 48 patient interviews, and 46 cognitive patient interviews, along with an expert opinion panel comprising plastic surgeons and other healthcare professionals. The scales were then tested on a sample of 2715 patients, with a response rate of 72%. The BREAST-Q reconstruction module has the following scales: satisfaction with breasts, outcome satisfaction, psychosocial well-being, sexual well-being, physical well-being, and chest and upper body satisfaction. In the BREAST-Q development sample (*n *= 1950), each scale fulfilled the Rasch and traditional psychometric criteria (including person separation index, 0.79–0.95; Cronbach’s alpha, 0.83–0.95; and test-retest reproducibility, 0.73–0.94).

## Results

**Fig. 1:**
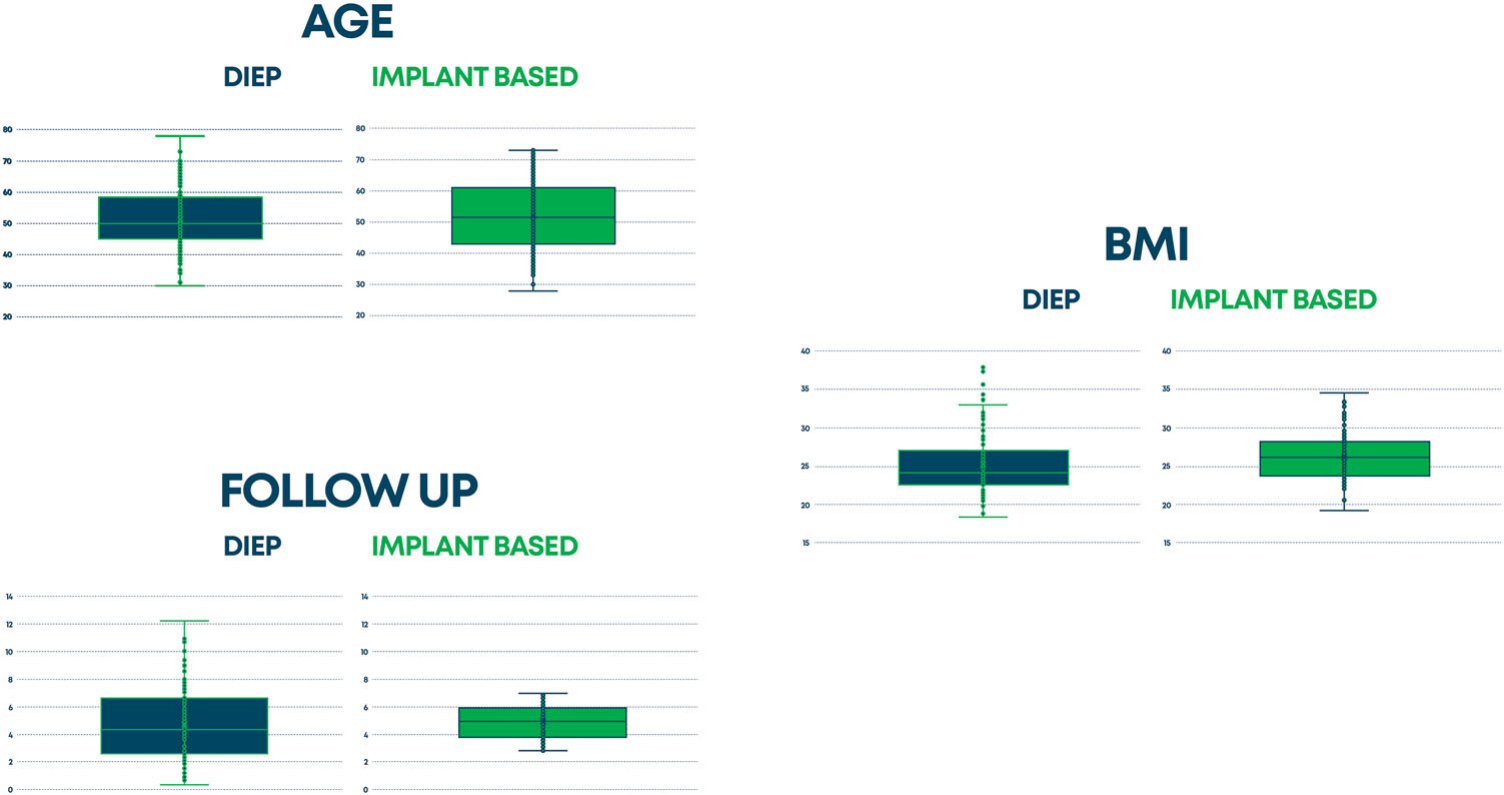
The average values of the BMI, age of patients, and follow-up of the two groups

**Fig. 2:**
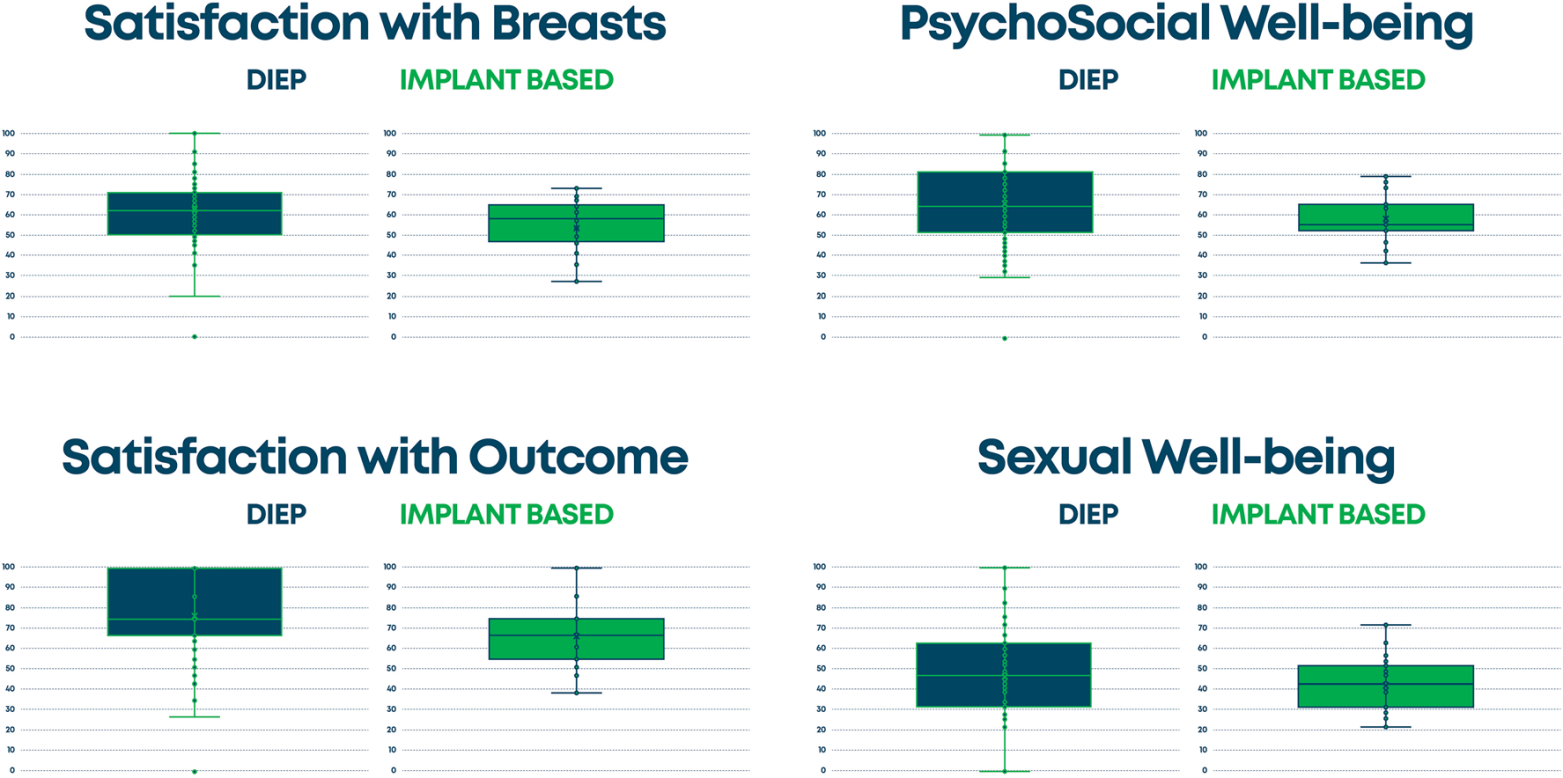
Results of the principal scales of BREAST-Q module

**Fig. 3:**
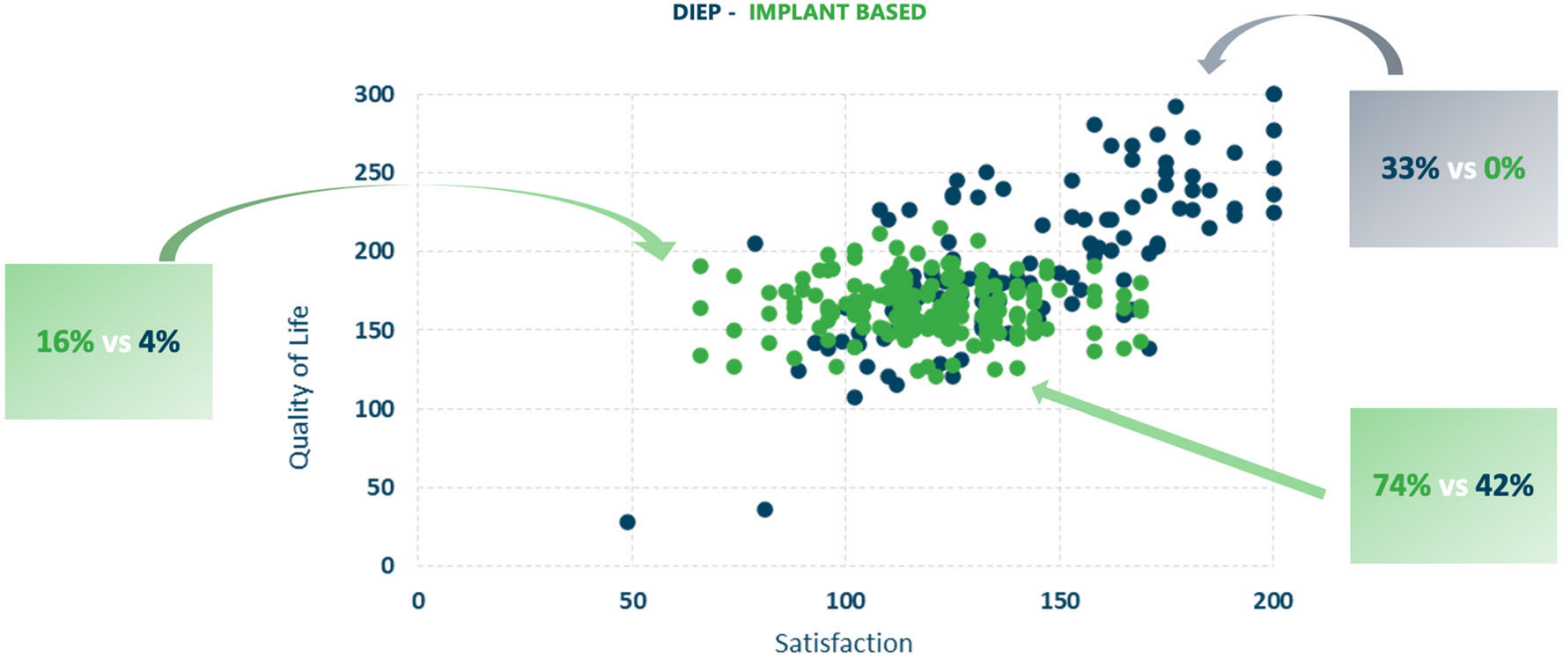
Quality of life and satisfaction of the two groups in base of the type of reconstruction

Of the 1125 patients involved, only 325 met the inclusion criteria and were enrolled in this study; specifically, 133 (41%) DIEP and 192 (59%) prosthetic reconstructions. The characteristics of the population studied (age, BMI, years since reconstruction, type of mastectomy, chemotherapy, radiotherapy, hormone therapy, comorbidities including diabetes, hypertension, and smoking) are shown in Table 1. Among those who underwent DIEP flap, 49 had a modified radical mastectomy, 11 had a radical mastectomy, 29 had a skin sparing mastectomy, 27 had a nipple mastectomy, and 7 patients had another type of mastectomy. For implant-based reconstruction, 30 patients underwent a modified radical mastectomy, 30 had a radical mastectomy, 30 had a skin sparing mastectomy, 50 underwent a nipple sparing mastectomy, 18 had a skin reducing mastectomy, and 34 patients had another type of mastectomy. There were 82.5% patients that underwent unilateral and 17.5% who underwent bilateral mastectomy and reconstruction. Pre-reconstructive therapies included radiotherapy in 48.3%, chemotherapy in 37.5%, and hormone therapy in 37.5%. Table 2 shows the results of all of the modules of BREAST-Q between the two groups with a statistical significance for the DIEP group (all scales with a *P* value < 0.001). In Figure 1, we summarized the average values of the BMI, age of patients, and follow-up of the two groups. For the age: First, there are no significant differences for the mean and variance of the two distributions (Levane's test is just > 0.05, 0.053 to be precise). The boxplot shows that the heterologous distribution has greater variability, the height of the boxplot is more marked (18 vs 13 years), as is the median (delta = 1.5). For follow-up: The tests do not reveal a significant difference between the means, while the variance is significant. At a glance, it is easy to see that the DIEP distribution is more variable than the implant-based (although the average and median are fairly aligned). DIEP patients had a lower BMI. The tests show that there are differences on average and the DIEP distribution is more variable. In Fig. 2, we summarized the results of the principal scales of BREAST-Q module: satisfaction with breast, psychosocial well-being, satisfaction with outcome, and sexual well-being in which the autologous group was always more satisfied. In Fig. 3, we represent the quality of life and satisfaction of the two groups in base of the type of reconstruction with a higher satisfaction and quality of life for DIEP. From Tables 3, 4, 5, 6, and 7, we reported results of all linear regression models with higher values for the DIEP group independently from predictors.

**Table 1:** Population data

Procedure type			
Characteristic	Autologous reconstruction (DIEP) (*n* = 133)*	Implant-based Reconstruction (*n* = 192)*	*P* value
Age, mean (SD)	51.3 (9.5)	51.9 (10.7)	0.622
Years after surgery, mean (SD)	4.7 (2.7)	4.8 (1.2)	0.632
BMI**, mean (SD)	25.2 (4.0)	26.2 (2.9)	0.017
*Laterality of reconstruction, number (%)*			
Unilateral	110 (82.7)	158 (82.3)	
Bilateral	23 (17.3)	34 (17.7)	0.923
*Mastectomy Type, number (%)*			
Modified radical	49 (36.8)	30 (15.6)	
Radical	11 (8.3)	30 (15.6)	
Skin sparing	29 (21.8)	30 (15.6)	< 0.001
Nipple sparing	37 (27.8)	50 (26.0)	
Other	7 (5.3)	52 (27.1)	
*Radiotherapy, number (%)*			
Yes, adjuvant	50 (37.6)	76 (39.6)	
Yes, neoadjuvant	11 (8.3)	20 (10.4)	0.698
No	72 (54.1)	96 (50.0)	
*Chemotherapy, number (%)*			
Yes, adjuvant	8 (6.0)	25 (13.0)	
Yes, neoadjuvant	32 (24.1)	57 (29.7)	0.035
No	93 (69.9)	110 (57.3)	
*Hormone Therapy, number (%)*			
Yes	46 (34.6)	76 (39.6)	
No	87 (65.4)	116 (60.4)	0.360
*Diabetes, number (%)*			
Yes	4 (3.0)	8 (4.2)	
No	129 (97.0)	184 (95.8)	0.586
*Hypertension, number (%)*			
Yes	33 (24.8)	56 (29.2)	
No	100 (75.2)	136 (70.8)	0.387
*Smoking status*			
Never smoker	62 (46.6)	96 (50.0)	
Previous smoker	34 (25.6)	42 (21.9)	0.724
Current smoker	37 (27.8)	54 (28.1)	

*The cell values may not total to the overall cohort size owing to missing data

**Calculated as weight in kilograms divided by height in meters squared

**Table 2:** Results of all of the modules of BREAST-Q between the two groups

Procedure type			
BREAST-Q	Autologous reconstruction (DIEP) (*n* = 133)*	Implant-based reconstruction (*n* = 192)*	*P* value
Satisfaction with Breast, mean (SD)	62.7 (16.2)	52.9 (12.1)	< 0.001
Satisfaction with Outcome, mean (SD)	77.7 (18.8)	66.5 (17.2)	< 0.001
Psychosocial well-being, mean (SD)	67.1 (20.4)	57.7 (11.9)	< 0.001
Sexual well-being, mean (SD)	52.6 (23.5)	42.4 (10.3)	< 0.001
Physical well-being: chest, mean (SD)	73.3 (16.6)	65.2 (9.5)	< 0.001

*The cell values may not total to the overall cohort size owing to missing data.

**Table 3:** Linear regression model: satisfaction with breasts

Variable	*B*	Standard error	*t*	*P* value
Procedure type (ref = IBR) DIEP	11.169	1.781	6.270	0.000
*Mastectomy type (ref = modified radical)*				
Radical	4.446	2.893	1.537	Ns
Skin sparing	0.658	2.478	0.265	Ns
**Nipple sparing**	**4.978**	**2.268**	**2.194**	**0.029**
Other	5.958	3.334	1.787	ns
*Laterality (ref = bilateral)*				
Unilateral	4.157	2.866	1.450	ns
Years after surgery	− 0.644	0.436	− 1.477	ns
*Radiotherapy (ref = none)*				
Adjuvant	− 2.444	1.722	− 1.420	ns
Neoadjuvant	2.166	2.759	0.785	Ns
*Chemotherapy (ref = None)*				
Adjuvant	2.617	2.731	0.958	Ns
Neoadjuvant	− 0.933	1.831	− 0.509	ns
*Hormonotherapy (ref = No)*				
Yes	1.274	1.639	0.777	ns
Age at interview	− 0.029	0.077	− 0.379	ns
BMI	− 0.202	0.248	− 0.814	Ns
*Smoking (ref = nonsmoker)*				
Previous smoker	− 0.703	2.001	− 0.351	Ns
Current smoker	− 1.523	1.879	− 0.810	ns
*Diabetes (ref = no)*				
Yes	− 3.651	4.196	− 0.870	ns
*Hypertension (ref = no)*				
Yes	− 1.539	1.779	− 0.865	ns

**Table 4:** Linear regression model: satisfaction with outcome

Variable	*B*	Standard error	*t*	*P* value
Procedure type (ref = IBR) DIEP	11.536	2.304	5.008	0.000
*Mastectomy type (ref = Modified Radical)*				
Radical	4.424	3.729	1.186	Ns
Skin sparing	1.264	3.198	0.395	Ns
**Nipple sparing**	**6.365**	**2.939**	**2.166**	**0.031**
Other	0.289	4.306	0.067	ns
*Laterality (ref = Bilateral)*				
Unilateral	− 1.229	3.688	− 0.333	ns
Years after surgery	− 0.746	0.562	− 1.328	ns
*Radiotherapy (ref = none)*				
Adjuvant	− 2.524	2.219	− 1.138	ns
Neoadjuvant	− 0.421	3.553	− 0.119	Ns
*Chemotherapy (ref = None)*				
Adjuvant	4.072	3.513	1.159	Ns
Neoadjuvant	1.665	2.362	0.705	ns
*Hormonotherapy (ref = No)*				
Yes	2.569	2.115	1.215	ns
Age at interview	0.186	0.100	1.861	ns
BMI	− 0.268	0.332	− 0.809	Ns
*Smoking (ref = nonsmoker)*				
Previous smoker	2.125	2.578	0.824	Ns
Current smoker	0.617	2.423	0.255	ns
*Diabetes (ref = no)*				
Yes	− 0.070	5.397	− 0.013	ns
*Hypertension (ref = no)*				
Yes	− 0.129	2.301	− 0.056	ns

**Table 5:** Linear regression model: psychosocial well-being

Variable	*B*	Standard error	*t*	*P* value
Procedure type (ref = IBR) DIEP	11.082	1.967	5.633	0.000
*Mastectomy type (ref = Modified Radical)*				
**Radical**	**6.921**	**3.203**	**2.161**	**0.032**
Skin sparing	− 2.468	2.722	− 0.907	Ns
**Nipple sparing**	**7.301**	**2.492**	**2.929**	**0.004**
Other	1.879	3.664	0.513	ns
*Laterality (ref = Bilateral)*				
Unilateral	− 0.093	3.150	− 0.029	ns
**Years after surgery**	**− 1.669**	**0.480**	**− 3.480**	**0.001**
*Radiotherapy (ref = none)*				
Adjuvant	2.513	1.892	1.328	ns
Neoadjuvant	3.260	3.071	1.061	Ns
*Chemotherapy (ref = None)*				
Adjuvant	4.462	3.000	1.487	Ns
Neoadjuvant	− 0.854	2.013	− 0.424	ns
*Hormonotherapy (ref = No)*				
Yes	1.013	1.802	0.562	ns
**Age at interview**	**0.232**	**0.085**	**2.727**	**0.007**
BMI	− 0.228	0.272	− 0.837	Ns
*Smoking (ref = nonsmoker)*				
Previous smoker	1.581	2.201	0.718	Ns
Current smoker	− 2.566	2.068	− 1.241	ns
*Diabetes (ref = no)*				
Yes	5.185	4.611	1.125	ns
*Hypertension (ref = no)*				
Yes	1.292	1.963	0.658	ns

**Table 6:** Linear regression model: sexual well-being

Variable	*B*	Standard error	*t*	*P* value
Procedure type (ref = IBR) DIEP	11.036	2.189	5.042	0.000
*Mastectomy type (ref = Modified Radical)*				
Radical	− 0.641	3.494	− 0.183	Ns
Skin sparing	− 2.319	3.031	− 0.765	Ns
Nipple Sparing	1.681	2.761	0.609	ns
Other	1.533	4.116	0.372	ns
*Laterality (ref = Bilateral)*				
Unilateral	5.444	3.513	1.550	ns
Years after surgery	− 0.873	0.528	− 1.651	ns
*Radiotherapy (ref = none)*				
Adjuvant	− 0.563	2.085	− 0.270	ns
Neoadjuvant	4.287	3.276	1.309	Ns
*Chemotherapy (ref = None)*				
Adjuvant	3.047	3.351	0.909	Ns
Neoadjuvant	− 1.917	2.221	− 0.863	ns
*Hormonotherapy (ref = No)*				
Yes	− 1.019	1.982	− 0.514	ns
Age at interview	− 0.030	0.095	− 0.315	ns
BMI	− 0.273	0.316	− 0.862	Ns
*Smoking (ref = nonsmoker)*				
Previous smoker	2.870	2.441	1.175	Ns
Current smoker	0.087	2.265	0.038	ns
*Diabetes (ref = no)*				
Yes	0.323	4.981	0.065	ns
*Hypertension (ref = no)*				
Yes	0.983	2.170	0.453	ns

**Table 7:** Linear regression model: physical well-being chest

Variable	*B*	Standard error	*t*	*P* value
Procedure type (ref = IBR) DIEP	10.164	1.652	6.154	0.000
*Mastectomy type (ref = modified radical)*				
Radical	4.687	2.673	1.754	Ns
Skin sparing	− 0.610	2.288	− 0.267	Ns
**Nipple sparing**	**4.454**	**2.106**	**2.115**	**0.035**
**Other**	**7.255**	**3.079**	**2.356**	**0.019**
*Laterality (ref = Bilateral)*				
**Unilateral**	**5.394**	**2.647**	**2.038**	**0.042**
Years after surgery	− 0.762	0.402	− 1.894	ns
*Radiotherapy (ref = none)*				
Adjuvant	1.300	1.591	0.817	ns
Neoadjuvant	3.645	2.550	1.429	Ns
*Chemotherapy (ref = None)*				
Adjuvant	3.308	2.522	1.312	Ns
Neoadjuvant	− 1.412	1.693	− 0.834	ns
*Hormonotherapy (ref = No)*				
Yes	1.675	1.518	1.104	ns
Age at interview	0.023	0.072	0.316	ns
BMI	0.051	0.231	0.221	Ns
*Smoking (ref = nonsmoker)*				
Previous smoker	− 0.421	1.851	− 0.227	Ns
Current smoker	− 2.731	1.738	− 1.571	ns
*Diabetes (ref = no)*				
Yes	− 1.067	3.875	− 0.275	ns
*Hypertension (ref = no)*				
Yes	0.573	1.652	0.347	ns

## Discussion

In the literature, there is a systematic review and meta-analysis comparing BREAST-Q data between autologous and implant-based breast reconstructions [16]. This systematic review and meta-analysis was performed to compare patient-reported outcomes of implant-based and autologous breast reconstruction. We found that autologous reconstruction yields a higher satisfaction with overall outcomes and breast. These findings can aid clinicians when discussing breast reconstruction options with patients. Only nine studies published in the literature are reported in this review and none for the Italian population. A comparative study on breast reconstruction with prosthesis or autologous should ideally be conducted in every country due to cultural issues and to have data from all countries regarding this type of surgery. Cultural influences are important and play a central role in the perception of the body. Furthermore, the use of BREAST-Q with all its modules needs to have as much feedback as possible for the cultural adaptation of the translation. Alshammari [17] from Saudi Arabia concluded the paper saying that, among the 61 patients studied, there was no significant difference in satisfaction between the autologous breast reconstruction and implant-based reconstruction group; however, this study was limited by a small sample with a short follow-up period, but it remains a study from the Arabic population. Dean [18], with a population from Australia, concluded their paper by saying that breast reconstruction is highly effective in improving the well-being of women undergoing mastectomy and that BREAST-Q is well suited for clinical effectiveness research and is easily incorporated into routine patient care. The same conclusion was made in the study by Lagendijk [19] from the Netherlands, who found that the scores of BREAST-Q serve as a reference value for different types of surgery in the study population and enable prospective use of patient-reported outcome in shared decision-making. Liu [20], who studied a cohort of 119 patients from China, concluded that the majority of patients in their study were most satisfied with the microsurgical abdominal flap breast reconstruction using BREAST-Q. McCarthy [21] conducted a study on 308 patients from the USA and concluded that immediate autogenous tissue reconstruction experience results in significantly less chest and upper body morbidity than in those who undergo either mastectomy with implant-based reconstruction or mastectomy alone. Moberg [22] from Norway concluded that women who underwent autologous-tissue breast reconstruction were more satisfied with the overall outcome than those who underwent implant-based breast reconstruction. Pirro [23] from the Czech Republic found that 65 patients who underwent autologous-tissue reconstruction had better satisfaction and outcomes with the reconstructed breast, while both techniques appear to equally improve psychosocial well-being, sexual well-being, and chest satisfaction. Moreover, the group of Santosa [24] from USA concluded that patients who underwent autologous reconstruction were more satisfied with their breasts and had greater psychosocial well-being and sexual well-being than those who underwent implant reconstruction. Weichman [25] from Germany affirmed in the conclusions that in their sample, the microsurgical breast reconstruction is efficacious in patients with a body mass index less than 22 kg/m and, when compared with prosthetic reconstruction, results in higher satisfaction with breasts. Another study [26] which is not included in the first review that we cited because the authors did not use the BREAST-Q but analyzed the Assessment of Outcomes and Healthcare Resource Utilization After Immediate Breast Reconstruction Comparing Implant- and Autologous-based Breast Reconstruction, found that complications and secondary breast procedures, including unplanned revisions, after breast reconstruction were common and varied by reconstructive modality, and the frequency of these secondary procedures adds substantial healthcare charges to the care of the breast reconstruction patient. Hu, et al. [27] (USA) compares 110 expander/implant and 109 transverse rectus abdominis myocutaneous reconstructions and they concluded that in the long term, TRAM patients had significantly greater esthetic satisfaction compared to those that had an expander/implant performed. One of the most important published studies about this topic is by Nelson et al. [28] (USA) that consisted of a cohort of 3268 patients, including 336 who underwent autologous breast reconstruction and 2932 that had implant-based breast reconstruction. This study presented the largest prospective examination of patient-reported outcomes in post-mastectomy reconstruction to date. Patients who opted for an autologous breast reconstruction had significantly higher satisfaction with their breast and quality of life at each assessed time point, but IBR patients had stable long-term satisfaction and quality of life postoperatively. All of these studies are important because they highlight two important points: (1) breast reconstruction is an integral part of the treatment after mastectomy and represents the surgical part that improves the quality of life of patients and (2) the choice of the technique is important and must be based on precise criteria and according to patient characteristics; moreover, reconstruction with the autologous technique remains the most satisfactory in the long term [29–31]. There is no one better technique than another, but we can certainly say that autologous techniques are better perceived by patients [32]. It would be excellent to discuss the bioethical concepts of a breast prosthetic device and its role in breast reconstruction to understand the real perception that one has of this device that is not originally part of the body [33]. Our study is the first to be carried out on an Italian population, and it contributes to increasing the case history regarding the comparison between autologous techniques and the use of prostheses and their impact on the patient’s quality of life. There have not been any other studies conducted in our country concerning this topic. Therefore, our contribution is fundamental to communicate that autologous techniques are also perceived as the most satisfactory in the long term in our population.

## Conclusions

This is the first study performed on the Italian population that compares autologous surgical techniques with the implantation of breast implants. In this population, DIEP is considered the technique that leads to the highest satisfaction in all BREAST-Q scores. Each country should conduct a study on this topic because the perception of one's body could be influenced by cultural factors and it would be interesting to analyze the case history of each country that deals with this type of surgery.
